# Design of a randomized controlled trial of a partnership-based, needs-tailored self-management support intervention for post-treatment breast cancer survivors

**DOI:** 10.1186/s12885-020-06861-x

**Published:** 2020-05-01

**Authors:** Soo Hyun Kim, Yu Hyeon Choe, Ah Reum Han, Gwui Jeong Yeon, Gyeong Hee Lee, Bo Gyeong Lee, Young Up Cho, Seho Park, Moon Hee Lee

**Affiliations:** 1 Department of Nursing, Inha University, 100 Inha-ro, Michuhol-gu, Incheon, 22212 South Korea; 2 College of Nursing, The Research Institute of Nursing Science, Daegu Catholic University, Daegu, South Korea; 3 Department of Surgery, Ilsan Women’s & Children’s Hospital, Cha University, Goyang, South Korea; 4 Division of Breast Surgery, Department of Surgery, Yonsei University College of Medicine, Seoul, South Korea; 5 Division of Hematology-Oncology, College of Medicine, Inha University, Incheon, South Korea

**Keywords:** Breast cancer, Randomized controlled trial, Self-management, Empowerment, Self-efficacy, Health-related quality of life

## Abstract

**Background:**

Self-management is becoming essential for breast cancer survivors, but evidence about the effectiveness of self-management support (SMS) intervention is lacking. To address this issue, we developed a theory-based SMS intervention, the ‘EMPOWER’, aimed at empowering breast cancer survivors. Here we describe the rationale of the intervention and its development.

**Methods:**

The conceptual framework of this study is the Chronic Care Model, which posits that SMS can influence patient-provider relationships and ultimately improve health outcomes. We will conduct a multi-center, 2-armed randomized controlled trial to assess the effectiveness of EMPOWER among post-treatment breast cancer survivors in South Korea. The trial will include 94 women who completed primary breast cancer treatment within the last 6 months. Participants will be randomly assigned to the intervention group or the wait-list control group (1:1). The intervention group will receive a 7-week partnership-based and needs-tailored SMS intervention via telephone counseling. The primary outcome is empowerment. The secondary outcomes include self-efficacy for post-treatment self-management behaviors, mental adjustment, psychological distress, and health-related quality of life (HRQOL). Data will be collected by self-reported questionnaire at baseline, post-intervention, and 3-month follow-up.

**Discussion:**

We believe that the EMPOWER intervention could improve HRQOL of post-treatment breast cancer survivors by enhancing their empowerment. If found successful, it could aid clinicians engaged in the long-term care of breast cancer survivors.

**Trial registration:**

Clinical Research Information Service, KCT0004794. Registered 5 March 2020.

## Background

Breast cancer is one of most common cancers among women worldwide. Due to advances in early detection and treatment, approximately 90% of women with breast cancer survive at least 5 years after diagnosis [1]. At some point after treatment, breast cancer may be considered a chronic illness [2].

Breast cancer survivors (BCS) face the numerous health challenges that are associated with a complex chronic condition, including managing persisting symptoms, identifying signs and symptoms of progression, accessing needed information and support, and making healthy lifestyle changes [3, 4]. Shouldering the responsibility for self-management (SM) behaviors can help survivors live well [5]. Unfortunately, cancer survivors in general report feelings of vulnerability and often lack the confidence to initiate the actions required to recover after their treatment [6]. This has led to increasing calls for better SM enablement.

The Chronic Care Model (CCM) is one suggested model for cancer survivorship care [7]. Introduced by Wagner and colleagues [8] in 1998, it was designed to improve the management and health outcomes of individuals with chronic illnesses. In the CCM, the essential element of good care is a productive interaction between informed, motivated patients and a prepared practice team [8]. This interaction can be influenced by 6 components of the CCM [the health system, community resources, self-management support (SMS), delivery system design, decision support, and clinical information systems]. Of those 6 components, SMS has been featured as a key component for assuring quality healthcare [9].

SMS refers to support of an individual’s ability to manage the symptoms, treatment, and physical, psychosocial, and lifestyle changes inherent in living with a chronic condition [8]. The application of SMS intervention among cancer survivors is increasing, and several BCS studies report its efficacy for health outcomes [10–15]. Types of SMS interventions are various and include distress management [10], uncertainty management [11–13], coping [14], and lifestyle management [15]. SMS interventions can significantly improve cancer knowledge [12, 13], cognitive reframing [11–13], self-efficacy [10, 15], and health-related quality of life (HRQOL) [15], and it can decrease psychological distress [10, 11]. Significance has not been demonstrated, however, for SMS effects regarding empowerment [10], patient-provider communication [11, 13], and social support [12], thus rendering the full efficacy of SMS intervention incomplete.

Managing everyday problems brought about by cancer and/or its treatment is likely to be enhanced by a collaborative partnership between patients and health care providers, all of whom are considered co-equal experts of the condition, albeit from different perspectives [16]. Such a collaborative approach can delineate how health care providers can support patients in their SM behaviors [6]. Given the importance of a productive interaction between patients and health care providers in chronic care, an evaluation of relationship-related outcomes such as empowerment is necessary.

In addition, the ‘one-size fits all’ approach for chronic disease SM may not be adequate for a chronic illness as complex as cancer. Several studies have shown that current healthcare systems do not meet the survivors’ needs [17, 18]. Many experts suggest that an individualized or tailored approach should be adopted in survivorship care planning [19]. Because, to the best of our knowledge, few studies have incorporated needs-tailored SMS intervention among BCS, we developed EMPOWER (Partn**E**rship-based, tailored self-**M**anagement support **P**rogram f**O**r **W**omen with breast canc**ER**)—a partnership-based, needs-tailored SMS intervention for BCS who completed their primary treatment. The goal of EMPOWER is to enhance empowerment and increase self-efficacy for SM behaviors, thereby improving health outcomes among post-treatment BCS. This paper describes the design and methodological plan for a randomized controlled trial (RCT) to evaluate the effectiveness of EMPOWER in post-treatment BCS.

### Hypotheses of the EMPOWER trial

The objective of this RCT is to test whether the EMPOWER intervention is effective, compared with a control group, in improving health outcomes (mental adjustment, psychological distress, and HRQOL) by enhancing empowerment and increasing self-efficacy for SM behaviors among post-treatment BCS.

## Methods/design

The EMPOWER trial’s study design and its intervention are in in concordance with the guidelines of the Consolidated Standards of Reporting Trials 2010 statement [20] and the standard protocol Items: Recommendations for Interventional Trials (SPIRIT) [21].

### Study design

**Fig. 1:**
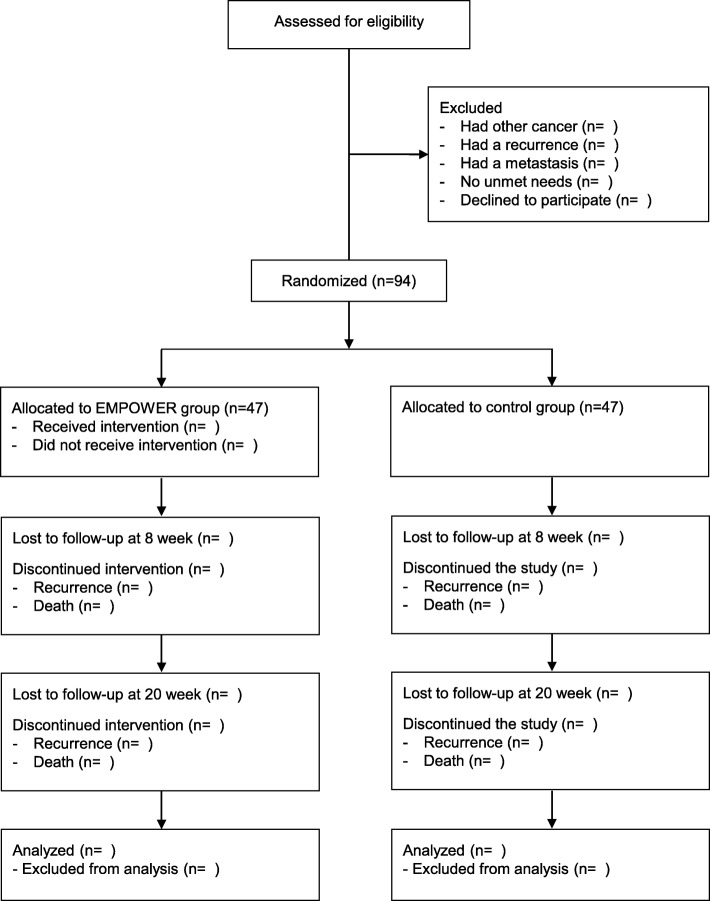
RCT flow chart. RCT, randomized controlled trial

**Fig. 2:**
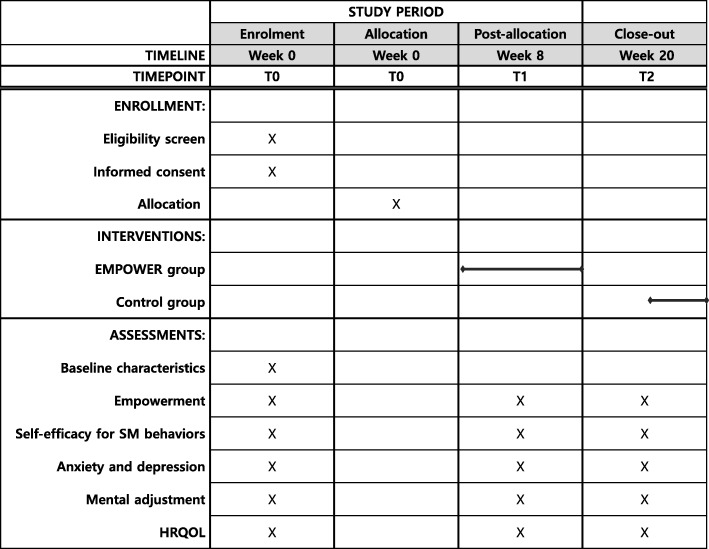
SPIRIT schedule of enrollment, interventions and assessment. HRQOL, health-related quality of life

This study is a 2-armed RCT designed to test the effects of EMPOWER vs a control intervention. A 7-week EMPOWER intervention will be assessed at baseline (T0), 8 weeks (T1), and 20 weeks (T2). Figure 1 shows a flow chart of the RCT; Fig. 2 shows the schedule of enrollment, interventions, and assessments. The study will be undertaken in South Korea’s two university hospitals—Yonsei Medical Center and Inha University Hospital. The Institutional Review Boards of both provided ethical approval. Written informed consent will be obtained from the participants.

### Participants of the study

Women will be eligible to participate if they 1) are aged 19 years or more, 2) were diagnosed histologically with breast cancer, 3) were treated with a curative cancer therapy (surgery, chemotherapy, or radiation therapy), 4) completed a primary cancer treatment within the previous 6 months (except for anti-hormone therapy and targeted therapy) 5) had two or more unmet needs in a post-treatment screening test covering 12 items of unmet needs concerning SM behaviors (i.e., follow-up visit, pain management, fatigue management, insomnia management, lymphedema management, exercise, diet, smoking cessation, alcohol consumption, stress management, return to work, and sexual activity), and 6) are able to use the telephone. Women will be excluded if they had a recurrence, a metastasis, or another cancer.

### Setting and procedure

Potential participants will be identified through physician-referral and self-referral. Physicians (YU Cho, S Park and MH Lee) will use electronic medical records (EMRs) to screen them for eligibility criteria related to diagnosis and treatment history and will tell potentially eligible women about the study. The researcher (YH Choe) will screen for more detailed eligibility criteria among women who are interested in participating in the study. Women who learned of our study through a flyer and are interested in participating can contact the researcher (YH Choe), who will screen them for eligibility via a telephone interview and will review medical information in the EMRs. Women who are finally eligible will meet with the researcher, and all participants will meet in the hospital and provide written informed consent.

Data will be collected via a self-reported questionnaire. After randomization, participants will complete the baseline assessment in a face-to-face interview. The researcher will contact participants via telephone 8 and 20 weeks after baseline and encourage them to complete a follow-up questionnaire via letter mail and send it back in an enclosed pre-addressed and stamped return envelope.

### Randomization

After the baseline assessment, we will use a computer-generated block randomization procedure (block size 4, 6, 8) in a 1:1 allocation ratio to randomize participants to either the EMPOWER group or the control group. Group assignments will be placed in sealed, sequentially numbered envelopes and opened by the participants. The recruiter will thereby be blinded to the study arm assignments of the participants. Because of the social nature of the intervention, however, participants cannot be blinded to the study arm assignments.

### Study groups

#### The EMPOWER intervention group

Based on the CCM, EMPOWER involves provider-participant partnerships. Such partnerships that help participants play a larger role in managing their post-treatment medical problems, thereby helping them reach their care goals [7]. SMS empowerment can extend to late and long-term treatment effects as well, helping survivors understand when to seek support and encouraging healthy lifestyle changes [6].

Participants in the EMPOWER group will receive a 7-week partnership-based, needs-tailored SMS intervention via telephone counseling (ten 15- to 20-min sessions, totaling 175 min). We extracted the intervention program contents from the conceptual SM framework in chronic illness [22], the qualitative data from a Korean BCS focus group interview regarding post-treatment SM needs [23] structured by 5 SM tasks (medical management, symptom management, lifestyle management, emotional management, and role management) and 21 specific topics (Table 1). An expert advisory team (3 surgeons, 1 advanced practice nurse, and 1 nursing professor) validated the final thematic structure.

**Table 1:** Thematic structure and contents of the EMPOWER intervention

SM task	SM education topic	SM skill training
Medical management	Follow-up after treatment	
	Late/long-term effects	
	Sign and symptoms of recurrence	
	Second cancer screening	
	Vaccination	
	Side effects of anti-hormone therapy (if applicable)	
Symptom management	Pain	√
	Peripheral neuropathy	
	Fatigue	√
	Insomnia	√
	Lymphedema	
Lifestyle management	Exercise	√
	Diet	√
	Smoking cessation	
	Alcohol restriction	
	Weight control	
Emotional management	Distress	√
Role management	Body image	
	Sexuality (if applicable)	
	Return to work (if applicable)	
	Recovery of self-confidence	

*SM* self-management

The EMPOWER intervention is subdivided into a 3-week SM education part and a 4-week SM skill training part (Table 2). At the end of the education part, participants will receive SM skill training in the topic of their choice. Currently, we have modules for the 6 topics (pain, fatigue, insomnia, exercise, diet, and distress) we have accumulated evidence for the intervention. Through participant choice, providers can tailor SM skill training to individual needs.

**Table 2:** Delivery of the EMPOWER intervention

Week	Session no.	Topic	Telephone time (min.)
1	1	SM education: Medical management	20
	2	SM education: Symptom management	20
2	3	SM education: Lifestyle management	20
	4	SM education: Emotional management	20
3	5	SM education: Role management	20
	6	1st SM skill training: Selected own topic	15
4	7	2nd SM skill training: Selected own topic	15
5	8	3rd SM skill training: Selected own topic	15
6	9	4th SM skill training: Selected own topic	15
7	10	Discussion: Healthy future plan	15
		Total	175

Using a 96-page evidence- and theory-based workbook, master-level nurses will deliver the SM education and skill training by telephone. The education and action planning contents of the workbook were extracted from the Korean National Cancer Information Center [24], the National Comprehensive Cancer Network [25], and the Oncology Nursing Society [26]. During the education sessions, providers will exploit Badura’s self-efficacy sources [27], such as verbal persuasion, vicarious experience, mastery, and physiological states. The workbook, for example, includes various vicarious experiences leading, for example, to success in weight management, exercise performance, and work resumption. During the SM skill training sessions, providers will present Lorig and Holman’s SM skills of problem solving, decision-making, taking action, forming partnerships, and utilizing resources [22]. The training uses a structured module according to a weekly plan (Table 3). The participant workbook is composed of a structured format that includes problem identification, goal setting, action planning, resource identification, and action monitoring. Facilitation of provider-participant partnerships is provided by a telephone counseling module that uses motivational interviewing principles (i.e., open questions, affirmation, reflective listening, and summary reflections) [28].

**Table 3:** Weekly contents of 6 self-management skill training modules

Module	Goal	Week 1	Week 2	Week 3	Week 4	Week 5
Pain	Able to verbally express own pain and practice according to pain Mx plan.	Weekly goal: Understand the pattern of pain (severity, location, quality, duration, etc.). Action plan: Making a pain diary	Weekly goal: Learn ways of pain Mx based on each pain type. Action plan: Making a pain diary.	Weekly goal: Practice pain Mx plan. Action plan: Making a pain diary.	Weekly goal: Practice pain Mx plan. Action plan: Making a pain diary.	Weekly goal: Plan future life.
Fatigue	Able to identify the cause of own fatigue and practice according to fatigue Mx plan.	Weekly goal: Identify the cause and pattern of fatigue. Action plan: Self-assessment of fatigue.	Weekly goal: Learn ways of fatigue Mx. Action plan: Making a fatigue diary.	Weekly goal: Practice fatigue Mx plan. Action plan: Making a fatigue diary.	Weekly goal: Practice fatigue Mx plan Action plan: Making a fatigue diary.	Weekly goal: Plan future life.
Insomnia	Able to identify pattern of own insomnia and practice according to insomnia Mx plan.	Weekly goal: Identify insomnia pattern. Action plan: Making a sleep diary.	Weekly goal: Learn ways of insomnia Mx (stimulation control). Action plan: Making a sleep diary.	Weekly goal: Practice insomnia Mx plan. Action plan: Making a sleep diary.	Weekly goal: Practice insomnia Mx plan. Action plan: Making a sleep diary.	Weekly goal: Plan future life.
Exercise	Able to establish individualized exercise goal and practice according to exercise plan.	Weekly goal: Understand 4 elements of exercise (frequency, intensity, timing, type). Action plan: Making an exercise contract.	Weekly goal: Establish individualized exercise goal. Action plan: Making an exercise diary.	Weekly goal: Practice the exercise plan. Action plan: Making an exercise diary.	Weekly goal: Practice the exercise plan. Action plan: Making an exercise diary.	Weekly goal: Plan future life.
Diet	Able to establish individualized diet goal and practice according to diet plan.	Weekly goal: Understand importance of balanced diet. Action plan: Making a diet contract.	Weekly goal: Establish individualized diet goal. Action plan: Making a diet diary.	Weekly goal: Practice diet plan. Action plan: Making a diet diary.	Weekly goal: Practice diet plan. Action plan: Making a diet diary.	Weekly goal: Plan future life.
Distress	Able to identify types of distress and practice according to emotional Mx plan.	Weekly goal: Identify level, cause, type of distress. Action plan: Completing NCCN distress thermometer.	Weekly goal: Learn ways of emotional Mx. Action plan: - Anxiety: Practicing abdominal breathing - Depression: Exercise or meditation (preferred)	Weekly goal: Practice emotional Mx plan. Action plan: - Anxiety: Practicing abdominal breathing - Depression: Exercise or meditation (preferred)	Weekly goal: Practice emotional Mx plan. Action plan: - Anxiety: Practicing abdominal breathing - Depression: Exercise or meditation (preferred)	Weekly goal: Plan future life

*Mx* management, *NCCN* National Comprehensive Cancer Network

### The control group

Participants in the control group will receive a 51-page education book whose content is the same as Part 1 of the intervention workbook. It includes SM strategies after cancer treatment but excludes SM skill training. At the end of the study, the control group can request the intervention.

### Study outcomes

The overview of the psychometric properties of primary and secondary outcome measures is presented in Table 4.

**Table 4:** EMPOWER study outcomes

Outcome	Measurement used	Measurement description	Psychometric properties
**Primary outcome**			
Empowerment	Empowerment Scale for Women with Breast Cancer	30 items and 3 factors. Factors include ‘intrapersonal factor’ (14 items), ‘interactional factor’ (8 items), and ‘behavioral factor’ (8 items). 5-point Likert scale. High score indicates higher empowerment.	Goodness of fit of the final research model was very appropriate as shown by χ^2^/df = 1.86, TLI = 0.90, CFI = 0.92, SRMR = 0.06, and RMSEA = 0.05. Criterion validity was evaluated by total correlation with the Cancer Empowerment Questionnaire 0.78. Cronbach’s alpha for total items was 0.93, and test-retest reliability was 0.69 [29].
**Secondary outcomes**			
Self-efficacy	Korean version of the Cancer Survivors’ Self-Efficacy Scale	10 items and 2 factors. Factors include ‘Self-efficacy for managing health problems’ (5 items) and ‘self-efficacy for seeking help and support’ (5 items). 10-point Likert scale. Higher score indicates higher self-efficacy.	Construct validity was evaluated with general self-efficacy (0.511), anxiety (−0.596), depression (− 0.554) and health-related quality of life (0.586). Cronbach’s alpha of total scale and subscales was 0.86–0.92 [30].
mental adjustment	The Mini-Mental Adjustment to Cancer	29 item and 4 factors. Factors include Helpless-Hopeless (HH), Anxious Preoccupation (AP) Positive Attitude (PA), Cognitive Avoidance (CA), and Fighting Spirit (FS).	Construct validity was evaluated with each of anxiety and depression subscales of HADS, AP (0.63, and 0.58), HH (0.54, and 0.59), FS (−0.30, and − 0.37), and PA (− 0.19, and − 0.23). Cronbach’s alpha was 0.50–0.86 and test–retest reliability was 0.68–0.88 [32].
Anxiety and depression	Hospital Anxiety and Depression Scale	14 item and 2 subscales. Subscales are ‘an anxiety (HADS-A)’ and ‘a depression (HADS-D)’. 4-point Likert scale. Higher score indicates greater anxiety or depression.	Construct validity of HADS-D was evaluated with Beck Depression Inventory 0.80, and HADS-A with Self-Rating Anxiety Scale was 0.79. Items of the HADS-A and corrected item total score was 0.55 and HADS-D was 0.47. Cronbach’s alpha for total items was 0.89 and 0.86 [35].
HRQOL	36-Item Short-Form Survey	36 items, 8 subscales and 2 domains. Domains include ‘physical component’ and ‘mental component’. Each subscale is scored 0 to 100. Higher score indicates better functioning and well-being.	The SF-36 has been validated in South Korea. Cronbach’s alpha was 0.89 for physical component, 0.87 for mental component, and 0.93 for total score [37].

*CFI* comparative fit index, *HRQOL* health-related quality of life, *RMSEA* root mean square error of approximation, *SRMR* standardized root mean square residual, *TLI* Tucker-Lewis Index

### Primary outcome

We will evaluate empowerment using the Empowerment Scale for Women with Breast Cancer [29]—a 30-item self-report instrument consisting of intrapersonal factors (14 items), interactional factors (8 items), and behavioral factors (8 items). Scored on a 5-point Likert scale, a high score indicates higher empowerment. The scale has shown good validity and reliability [29].

### Secondary outcomes

We will measure self-efficacy for SM among BCS using a Korean version of the Cancer Survivors’ Self-Efficacy Scale (CSSES-K) [30]. The original version of CSSES is an 11-item questionnaire assessing cancer survivors’ cancer-related self-efficacy [31]. The CSSES-K is a 10-item, 2-factor questionnaire. The factors are ‘self-efficacy for managing health problems’ (5 items) and ‘self-efficacy for seeking help and support’ (5 items) [30]. Each item is rated on a 10-point scale from 1 (not at all confident) to 10 (totally confident); a higher score indicates higher self-efficacy. The original version of CSSES has a good reliability and validity [31], thus the CSSES-K has adequate internal consistency (Cronbach’s alpha = 0.86–0.92) [30].

We will evaluate mental adjustment using a Korean version of Mini-Mental Adjustment to Cancer (Mini-MAC) [32]. The original Mini-MAC is a 29-item self-rating questionnaire and includes 5 factors: 4 for Fighting Spirit (FS), 8 for Help-Hopeless (HH), 8 for Anxious Preoccupation (AP), 5 for Fatalism (FA), and 4 for Cognitive Avoidance (CA) [33]. The Korean version of Mini-MAC uses a 4-point Likert scale and includes 4 factors—8 items for HH, 8 for AP, 4 for CA, and 9 for Positive Attitude (PA). The Cronbach’s alpha coefficients of the Korean version of Mini-MAC are 0.50–0.86, and test-retest coefficients are 0.68–0.88 [32].

Anxiety and depression will be assessed using the Hospital Anxiety and Depression Scale (HADS) [34]. HADS is a 14-item self-report instrument assessing symptoms of anxiety and depression that reflects 2 subscales, with 7 items for depression and 7 items for anxiety. Each item is scored from 0 to 3, with higher scores indicating more distress. The Korean HADS has been validated and has shown good validity and reliability [35].

We will measure HRQOL using the 36-item Short-Form Health Survey (SF-36) [36]—a 36-item questionnaire that consists of 2 domains (physical and mental) and 8 subscales (functioning, physical role functioning, bodily pain, general health, vitality, social role functioning, emotional role functioning, and mental health). Each subscale is scored from 0 to 100, with higher scores indicating better functioning and well-being. The SF-36 has been translated into Korean and shown good validity and reliability [37].

### Sample size calculation

Based on the primary outcome ‘empowerment’, at least 78 patients will be required using an effect size of 0.65 [38], a power of 0.8, and an alpha less than 0.05. Assuming an estimated dropout of 20%, 47 participants will be needed in each group (a total of 94 participants).

### Statistical analysis

We will describe the characteristics of the study participants using frequency and percentage for categorical variables and means and standard deviations for continuous variables. To compare baseline characteristics between the two groups, we will use independent *t*-tests for normally distributed continuous variables, the Mann-Whitney test for non-parametric variables, and the chi square test for categorical variables. We will use linear mixed models to analyze the efficacy of EMPOWER and Cohen’s d to estimate effect size as large (≥0.80), moderate (0.5–0.79), or small (< 0.50) [39]. Missing data will be handled under the missing-at-random assumption. We will perform all analyses using IBM SPSS Statistics version 25.0 (IBM, Armonk, NY, USA) according to the intention-to-treat principle and consider a *p*-value < 0.05 significant.

## Discussion

We developed the EMPOWER intervention with the intention of improving health outcomes by enhancing empowerment and increasing self-efficacy in post-treatment BCS, thus facilitating successful transition from hospital-based survivorship care to SMS care. Expected outcomes include decreased psychological distress, better mental adjustment, and improved HRQOL. If successful, this study will provide evidence that SMS may be an important survivorship care model.

EMPOWER has several strengths. First, it is theory-based; we applied self-efficacy theory [27], Lorig and Holman’s model [22], and motivational interviewing technique [28] for development and delivery of the intervention. EMPOWER’s main mechanism will be to facilitate partnership between provider and participant and increase self-efficacy of the participant. Second, EMPOWER is needs-tailored. Although substantial BCS have unmet needs after cancer treatment [40, 41], there is a lack of interventions that meet their supportive care needs in a personalized manner. We believe that EMPOWER’s Part 2 intervention (4-week SM skill training for participant’s chosen topic) will work as a needs-based tailored intervention. Third, EMPOWER’s SM skill training protocol is evidence-based. We developed action-planning protocols for each SM topic based on interventions that were already proven effective.

EMPOWER has also several weaknesses. First, it can be resource intensive because it is delivered by a well-trained nurse via telephone. That burden could be reduced, however, by delivering the Part 1 intervention (SM education) by an app or on the web. Second, SM skill training is available for only 6 topics (management of pain, fatigue, insomnia, distress, exercise, and diet). As more evidence becomes available, other SM topics could be developed. Third, generalization to a wider setting and in other countries must be done with caution because the prioritized SM topics were chosen by the Korean population.

## Conclusion

EMPOWER is a theory-based SMS intervention unique in its provider-partnership and needs-tailored approach. If found successful, the EMPOWER trial will offer insights into how clinicians can engage in chronic care for BCS after treatment using SMS intervention.

## Data Availability

Not applicable.

## Abbreviations

BCS: breast cancer survivors
CCM: Chronic Care Model
CSSES-K: Korean version of the Cancer Survivors’ Self-Efficacy Scale
EMR: Electronic medical record
HADS: Hospital Anxiety and Depression Scale
HRQOL: Health-related quality of life
Mini-MAC: Mini-Mental Adjustment to Cancer
RCT: Randomized controlled trial
SF-36: 36-item Short-Form Health Survey
SM: Self-management
SMS: Self-management support
SPIRIT: Standard protocol Items: Recommendations for Interventional Trials
